# Potential of Stem Cell-Based Therapy for Ischemic Stroke

**DOI:** 10.3389/fneur.2018.00034

**Published:** 2018-02-06

**Authors:** Hany E. Marei, A. Hasan, R. Rizzi, A. Althani, N. Afifi, C. Cenciarelli, Thomas Caceci, Ashfaq Shuaib

**Affiliations:** ^1^Biomedical Research Center, Qatar University, Doha, Qatar; ^2^Department of Mechanical and Industrial Engineering, Qatar University, Doha, Qatar; ^3^Institute of Cell Biology and Neurobiology (IBCN), Italian National Council of Research (CNR), Rome, Italy; ^4^Qatar Biobank, Doha, Qatar; ^5^Institute of Translational Pharmacology (CNR), Roma, Italy; ^6^Biomedical Sciences, Virginia Tech Carilion School of Medicine and Research Institute, Roanoke, VA, United States; ^7^Neurosciences Institute, Academic Health System, Hamad Medical Corporation, Doha, Qatar; ^8^University of Alberta, Edmonton, AB, Canada

**Keywords:** stem cell, mesenchymal stem cell, neural stem cell, induced pluripotent stem cells, ischemic stroke

## Abstract

Ischemic stroke is one of the major health problems worldwide. The only FDA approved anti-thrombotic drug for acute ischemic stroke is the tissue plasminogen activator. Several studies have been devoted to assessing the therapeutic potential of different types of stem cells such as neural stem cells (NSCs), mesenchymal stem cells, embryonic stem cells, and human induced pluripotent stem cell-derived NSCs as treatments for ischemic stroke. The results of these studies are intriguing but many of them have presented conflicting results. Additionally, the mechanism(s) by which engrafted stem/progenitor cells exert their actions are to a large extent unknown. In this review, we will provide a synopsis of different preclinical and clinical studies related to the use of stem cell-based stroke therapy, and explore possible beneficial/detrimental outcomes associated with the use of different types of stem cells. Due to limited/short time window implemented in most of the recorded clinical trials about the use of stem cells as potential therapeutic intervention for stroke, further clinical trials evaluating the efficacy of the intervention in a longer time window after cellular engraftments are still needed.

## Introduction

The number of stroke-related deaths is increasing and stroke remains one of the major causes of deaths and disability worldwide (1, 2). Between 1990 and 2010, the global incidence rate of stroke seemed to be stable, while other parameters such as the incidence of first stroke, prevalence of stroke, disability-adjusted life-years lost due to stroke, and the number of stroke-related deaths increased by 68, 84, 12, and 26%, respectively (1). Differences between rates and numbers might reflect variations in population structure, increase in life expectancy, and the global improvement of health care services.

Two main types of stroke are recognized: ischemic and hemorrhagic stroke. Ischemic stroke accounts for over 80% of the total number of strokes. Thrombolysis and/or thrombectomy is the only validated therapeutic strategy for ischemic stroke (3, 4). Neurorestorative stem cell-based therapy is currently a major priority for stroke research (5, 6). Following ischemic events an inflammatory cascade, is initiated eventually leading to damage of brain tissue.

## Different Cellular Sources Used for Stem Cell-Based Therapy of Stroke

**Figure 1:**
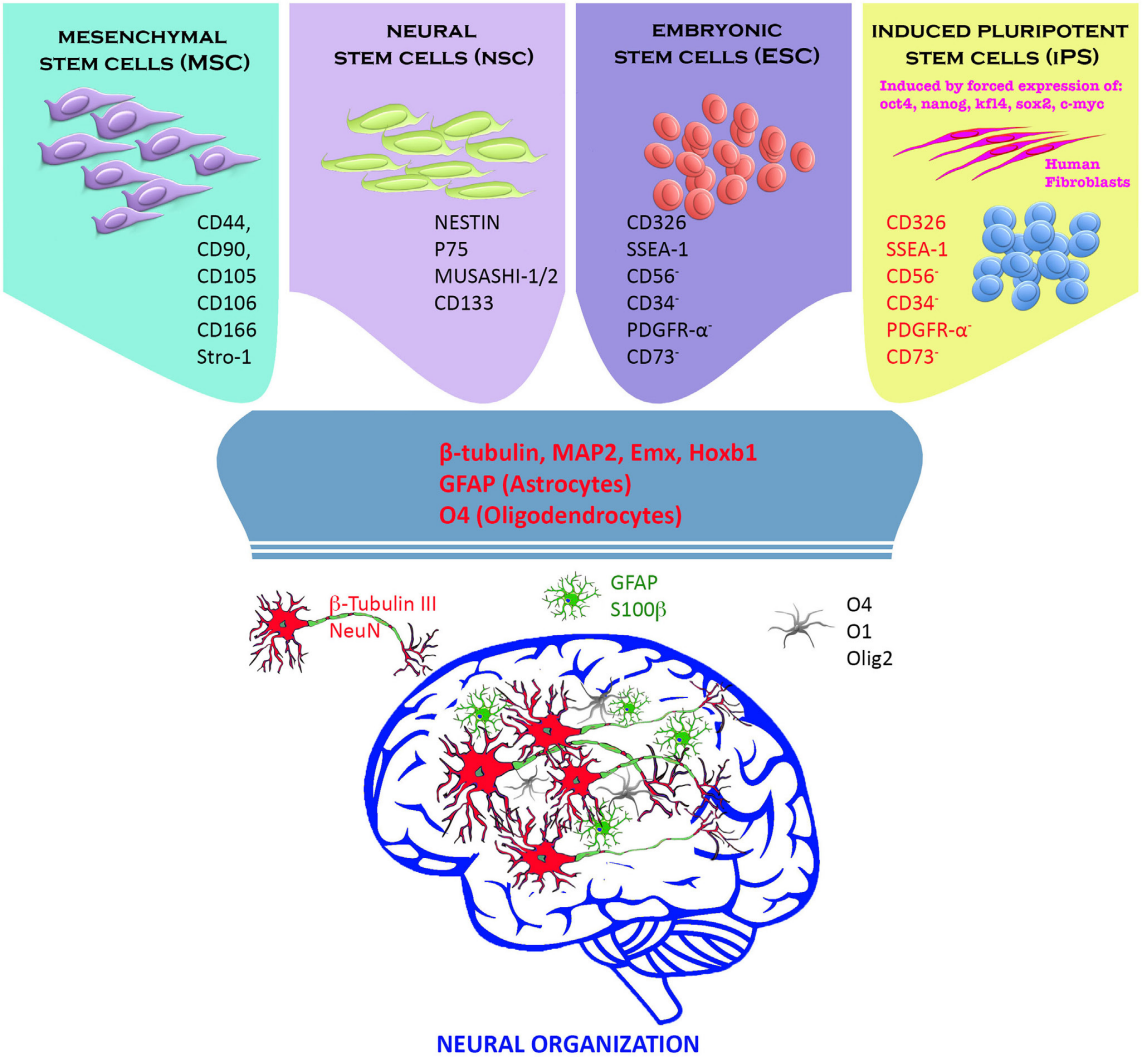
Stem cells and neural progenitor cells have been used to replace neural tissue death following a cerebral insult. Adult (mesenchymal and neural stem cells) and embryonic stem cells (ESCs) exhibited excellent differentiation capacity toward the neural phenotypes (neurons, oligodendrocytes, and astrocytes) *in vitro* and *in vivo*. In our view instead, induced pluripotent stem cells (iPSCs) constitute the greatest prospect for a future cell therapy. iPSCs are derived directly from the patient’s connective tissue through a small biopsy and exhibit the same properties of ESCs, overcoming the problems related to immune rejection, and bypassing the need for embryos. They can be generated in a patient-matched manner, implicating that each individual could have their own pluripotent stem cell line. Finally, iPSCs can be used in personalized drug discovery and to understand and deepen the patient-specific basis of disease (7–10).

The drastic damage to brain tissues following ischemic stroke includes not only destruction of a heterogeneous population of brain cell types, but also major disruption of neuronal connections and vascular systems. Several types of stem/progenitor cells such as embryonic stem cells (ESCs), neural stem/precursor cells, mesenchymal stem cells (MSCs), induced pluripotent stem cells (iPSCs), and induced neurons have been assessed as potential cellular-based therapy for stroke. The results of studies of these different cellular types are conflicting. In some studies, the engrafted cells survived, proliferated, differentiated, and restored lost neuronal and vascular elements. Other studies have shown only a limited neurorestorative ability on the part of transplanted cells. In the next section of this review, we elaborate on different stem cell types used for cellular-based therapy of stroke (Figure 1).

## Embryonic Stem Cells

Derived from the inner mass of blastocysts, ESCs are pluripotent cells having the ability to differentiate into all other body cells except those of the placenta (11). The regenerative capacity of ESC in stroke is related to their ability to give rise to different neuronal and glial elements forming the brain tissues (i.e., neurons, astrocytes, and oligodendrocytes) (12). Engrafted murine ESCs in cerebral tissue in an ischemic mouse model migrated toward damaged brain areas in the opposite cerebral hemisphere, restored histological and behavioral deficits (13), and repaired damaged synaptic connections associated with stroke lesions (14).

## Neural Stem/Precursor Cells

Neural stem cells (NSCs) are multipotent cells residing mainly in the subgranular zone of the dentate gyrus of the hippocampus (15), and in the subventricular zone of the brain’s third ventricle (16). The NSCs move from the subventricular zone into the rostral migratory stream and thence to the olfactory bulb where they differentiate into interneurons. Currently, NSCs are a hot research area for neurobiologists. Their ability to differentiate into different neuronal and glial elements that form the CNS make them a promising candidate for restoration of neuronal and behavioral damages associated with different CNS disorders including stroke.

The first attempt to use NSC for cell-based therapy of brain hypoxia was conducted in 1984 when embryonic brain cortex tissue was engrafted in a rat hypoxia model. The transplanted cells proliferated, established connections with host neurons, and improved electrophysiological performance (17, 18).

Embryonic NSCs engrafted into ischemic rat brains survived, migrated to the ischemic lesion, maturing into neurons (19, 20) as well as astrocytes and microglia (21); they restored impaired sensorimotor and spatial learning functions (22). In a macaque stroke model, engrafted NSCs partially differentiated into neurons, and survived up to 105 days (23).

## Mesenchymal Stem Cells

Mesenchymal stem cells can be derived from several tissue sources, including bone marrow, placenta, muscle, skin, dental pulp, adipose tissue, umbilical cord, and Wharton’s jelly (24, 25). The therapeutic potential of bone marrow MSC (BMSC) for stroke has been extensively assessed both at the preclinical and clinical levels. In an animal stroke model, transplantation of BMSC enhanced sensorimotor function (26), promoted synaptogenesis, stimulated nerve regeneration (27), decreased tissue plasminogen activator-induced brain damage (28), and mediated immunomodulatory effects (29).

At the clinical level, BMSCs appeared to be an attractive alternative that avoided ethical concerns related to the used of fetal cells. Several studies have revealed the feasibility and safety of BMSCs in clinical practice (30–32).

## Induced Pluripotent Stem Cells

Reprogramming of somatic cells such as fibroblasts and peripheral blood mononuclear cells through transduction of defined transcriptional factors (Oct3/4, Sox2, Klf4, and c-Myc) is currently becoming a standardized protocol (33, 34). The therapeutic potential of iPSCs in treating various CNS diseases (including stroke) has been addressed in previous studies (35). In comparison with ESCs, iPSCs have the advantage of sparing the damage induced by immune rejection, and avoiding the moral issue associated with the use of embryonic tissues (36). Engraftment of iPSCs in a cerebral ischemia model reduced infarct volume, ameliorated the neurological outcomes, and improved short-term sensorimotor recovery (37). Unfortunately, following engraftment, iPSCs formed teratomas in mouse brains (38, 39). The high propensity of iPSCs for teratoma formation is attributed to the expression of matrix metalloproteinase-9 and phosphorylated vascular endothelial growth factor receptor 2 (40).

One of the promising strategy for the use of iPSC to treat stroke is their ability to differentiate into NSC. Induced pluripotent stem cell-derived neural stem cells (iNSCs) are expected to provide multipotent, autologous cells for stroke cellular-based therapy. In ischemic pig stroke model, implantation of iPSC-derived iNSC was associated with improved recovery. Several mechanisms have been reported to play a role in the observed improvement including cell replacement, and neuroprotection. Others changes have been demonstrated based on the use of longitudinal multiparametric magnetic resonance imaging. These include reduction in the changes of brain metabolism, cerebral blood infusion, and integrity of the white matter. Such tissue recovery in review 8 was primarily attributed to alleviation of negative immune response, activation of neurogenesis, and enhanced neuronal protection. These observation strongly support the importance of iNSCs as a promising cellular source to be used for cell-based therapy of human stroke (41).

## Tumoreginic Potential of Pluripotent Cells

One of the major concern for the use of pluripotent stem cells (including ESCs and iPSCs) for treatment of ischemic brain injury is their potential to develop a tumor following engraftment. Several studies have reported the tumorigenic transformation of iPSC (38, 40, 42, 43) following their in transplantation. The existence of a small number of undifferentiated iPSCs even after prolonged differentiation of iPSC *in vitro* may trigger the formation of teratoma *in vivo*, and pose a great risk against their clinical application (44). Other factors might also contribute to the tumorigenic potential of iPSC including the transcriptional factors and virus vectors used during iPSC induction (45, 46). The role of the four Yamanaka reprogramming factors (Klf4, c-Myc, Oct4, and Sox2) in induction of teratoma had been suggested by some authors, and they were found to be strongly expressed in iPSC-derived tumors (38). The four factors have been demonstrated to be highly expressed in various cancer types (47–49), and MYC has been demonstrated to be a well-documented oncogene (50, 51). The expression of aforementioned genes has been associated with poor prognosis, and tumor progression (52). The role of these transcription factors in the tumorigenic potential of iPSC has been indirectly demonstrated where inhibition of the tumor suppressors in the p53 pathway was found to increase the reprogramming ability of Oct4, Klf4, and Sox2 (53). Elimination of the “unsafe” undifferentiated residual cells has been suggested to guard against the development of iPSC-associated teratoma. Toward this aim, several strategies such as magnetic-activated cell sorting and fluorescence-activated cell sorting (54) have been used. Other strategies to mitigate potential tumorigenic potential of engrafted pluripotent cells include the use of cytotoxic antibodies such as mAb 84 (55), use of virus-free iPSCs, and encapsulation of pluripotent stem cell-derived grafts (56) were also effective.

## Immunogenicity of Stem Cell-Based Therapy for Stroke

The potential of allogeneic stem cells in the treatment of stroke has been highlighted before. Savitz et al. (57) have tested the potential of fetal porcine in transplantation in patients with basal ganglia infarcts and stable neurological deficits. In a trial to suppress the immunorejection of the transplanted cells, patients were pretreated with anti-MHC1 antibodies with no immunosuppressive drugs. No adverse effects have been observed, while the fourth patient exhibited a deterioration in motor functions deficits 3 weeks after transplantation. Other side effects that might indicate rejection of engrafted cells were shown in the fifth patients who have developed seizures 1 week after transplantation. The study was terminated by the FDA after the inclusion of five patients. This study was the first that pointed out to the potential use of non-tumor cells in ischemic stroke patients.

## Mechanism of Action of Stem Cell-Based Therapy for Stroke

The potential mechanism(s) by which different types of engrafted stem cells help to restore lost neuronal function after stroke are still a matter of dispute. Several mechanisms have been demonstrated including cell replacement, trophic influences, immunomodulation, and enhancement of endogenous repair processes.

The mechanism by which the engrafted BMSCs exerts their beneficial actions is still under investigation. Whether or not the improvement occurred following transplantation of BMSCs is a primary concern, but their ability to replace dead or damaged neuronal and glial elements still needs further confirmation.

Release of soluble trophic factors and cytokines is suggested as one major mechanism by which NSC bring about improvement in post-stroke neurological function (58). A wide array of trophic and growth factors has been reported to be released from endogenous cells such as astrocytes and endothelial cells (59). These include VEGF/Flk1 and Ang-1/Tie2 (60), BDNF, nerve growth factor, VEGF, IGF-1, hepatocyte growth factor, and GDNF. These factors promote angiogenesis, stabilize vasculature, enhance cell survival proliferation and differentiation, promote neurogenesis, effect endogenous cell repair, trigger neuroblast proliferation, and trigger migration from SVZ and decreased apoptosis (61).

## Cell Replacement

Cell replacement involves the ability of engrafted cells to migrate, survive, proliferate, and finally differentiate into the various types of cells forming nervous tissue histo-architecture. These include neurons of different classes, oligodendrocytes (the myelin forming cells), and astrocytes. Following stroke or other neurological insults/disorders several neurodegenerative and inflammatory pathways are activated creating an inhospitable environment for engrafted cells. Astrocytes usually respond by extensive proliferation and formation of a glial scar (62) which renders the damaged area unsuitable for engrafted exogenous cells.

Based on the initial number of cells engrafted and the route of administration, the necessary first step in restoring damaged cellular elements following stroke is the migration of transplanted cells to damaged brain regions. This is usually achieved through the ability of engrafted stem/progenitors cells to target damaged regions (63) in response to different chemotactic signals of specific cytokines, such as the vascular cell adhesion molecule 1, stromal-derived factor 1, monocyte chemotactic protein-1, chemokine (C–C motif) ligand 2 (21).

## Clinical Trials

In a recent meta-analysis of stem cell therapies for patients with brain ischemia, Chen et al. (64) concluded that stem cell therapy significantly enhanced neurological functions and quality of life, but more investigation is required to provide more evidence to support clinical application of stem cell transplantation (64, 65).

In a very recent clinical trial, the safety and efficacy of autologous bone marrow mononuclear cells transplantation in stroke patients were assessed. The study suggests that a higher dose of BM-MNC (3 × 10^6^ or more) provided a better outcome in stroke patients (66).

In another recent clinical trial, improved neurological function with no tumor formation or adverse events was demonstrated following engraftment of an immortalized human neural stem-cell line (67).

A double-blind randomized placebo-controlled Phase III confirmatory clinical trial of intravenous infusion of autologous NSC derived from bone marrow of stroke patients resulting from cerebral infarction is currently under way (68).

To evaluate the safety and clinical outcomes of surgical transplantation of modified bone marrow-derived MSCs, cells were engrafted in 18 patients with stable, chronic stroke. This therapeutic paradigm was proven to be safe, and was associated with improvement in clinical outcome end points after 12 months (69). Nagpal et al. (70) investigated the use of autologous stem cell therapy for stroke survivors with chronic disability. The primary outcomes to be measured are safety and feasibility of intracranial administration of autologous human adult DPSC in patients with chronic stroke; as well as determination of the maximum tolerable dose in humans. Secondary outcomes to be assessed include estimation of the measures of effectiveness required to design a future Phase 2/3 clinical trial (70).

In summary, the conclusions of several preclinical studies have encouraged the translation of stem cell-based therapies at the clinical level. Several clinical studies related to the use of different types of stem cells for cell-based therapy of stroke have been conducted since 2005 using MSCs (30), MNC (32, 71), and NSCs (57, 72).

## Ongoing Clinical Trials

To the best of our knowledge, there are currently more than 53 clinical trials on the use of stem cell-based therapy for stroke. Most of them use MSCs isolated from different body tissues: umbilical cord, endometrial polyps, menstrual blood, adipose tissue, and bone marrow (73). Although use of autologous MSCs is the method of choice to guard against immune rejection, the long time frame needed to obtain sufficient numbers of MSCs from the patient’s own tissue (i.e., bone marrow), makes the use of “off-the-shelf” allogeneic MSC therapy more convenient. Manipulation of MScs to overexpress genes with potentially beneficial properties and the ability to rapidly release different trophic factors was found to enhance their therapeutic potential and effects (74). Administration of multipotent adult progenitor cells was safe and well tolerated in patients with acute ischemic stroke. Although no significant improvement was observed at 90 days in neurological outcomes with multipotent adult progenitor cells treatment, further clinical trials evaluating the efficacy of the intervention in an earlier time window after stroke (<36 h) are planned (75).

Different routes of administration have been used to deliver stem cells into the stroke patients, namely intra-arterial, intravenous, and intraparenchymal routs. An early subacute delivery of cells to reduce acute tissue injury and modify the tissue environment in a direction favorable to reparative processes (for example, by being anti-inflammatory, anti-apoptotic, and encouraging endogenous stem cell mobilization); the other exploring later delivery of cells during the recovery phase after stroke to modulate the local environment in favor of angiogenesis and neurogenesis. The former approach has generally investigated intravenous or intra-arterial delivery of cells with an expected paracrine mode of action and no expected engraftment within the brain. The latter has explored direct intracerebral implantation adjacent to the infarct. Several relevant trials have been conducted, including two controlled trials of intravenously delivered bone marrow-derived cells in the early subacute stage, and two small single-arm phase 1 trials of intracerebrally implanted cells (76).

## Future Perspectives and Conclusion

Stem-based therapy for ischemic stroke is still in its infancy. Several alternative approaches including the use of ESCs, MSCs, NSC, and iPSCs have been tried in hopes of improving the drastic neuronal and functional impairment that usually follows a stroke insult. The outcomes of various preclinical studies have been encouraging, with (in most cases) engrafted stem cells succeeding in bringing about neurofunctional improvements. The mechanism(s) by which different types of stem cells induce improvement are still under investigation. Cell replacement, bystander effects, neurotrophic influence, immune and inflammatory modulation are all among the suggested mechanisms. At the clinical level, most of the clinical trials have used MSCs or NSCs (whether wild-type, genetically modified to overexpress certain neurotrophic genes, or preconditioned with the intent to promote cell survival and differentiation following transplantation) engrafted into an ischemic brain region. Autologous cells (mostly bone marrow-derived MSCs) are used in most of the ongoing clinical trials, although there is a current trend that favors the use of “off-the-shelf” allogeneic MSC as a way to overcome the long time frame needed to obtain sufficient numbers of cells for transplant. Most current clinical trials aim to measure the safety and feasibility of intravascular and/or intracranial administration of autologous/allogeneic human adult stem cells in patients with chronic stroke and to determine the maximum tolerable dose. Secondary outcomes include estimation of the measures of effectiveness required to design a future Phase 2/3 clinical trial.

## Ethical Statement

This article does not contain any studies with human participants or animals performed by any of the authors.

## Author Contributions

ME: study concept and design. HA, AA, and AN: analysis and interpretation. RR: acquisition of data. CC and TC: critical revision of the manuscript for important intellectual content. AS: study supervision.

## Conflict of Interest Statement

The authors declare that the research was conducted in the absence of any commercial or financial relationships that could be construed as a potential conflict of interest.

## References

[1] Feigin VL Forouzanfar MH Krishnamurthi R Mensah GA Connor M Bennett DA Global and regional burden of stroke during 1990–2010: findings from the Global Burden of Disease Study 2010. Lancet (2014) 383:245–55. 10.1016/S0140-6736(13)61953-4 24449944 PMC4181600

[2] Johnston SC Mendis S Mathers CD. Global variation in stroke burden and mortality: estimates from monitoring, surveillance, and modelling. Lancet Neurol (2009) 8:345–54. 10.1016/S1474-4422(09)70023-7 19233730

[3] Del Zoppo GJ Saver JL Jauch EC Adams HP Expansion of the time window for treatment of acute ischemic stroke with intravenous tissue plasminogen activator a science advisory from the American Heart Association/American Stroke Association. Stroke (2009) 40:2945–8. 10.1161/STROKEAHA.109.192535 19478221 PMC2782817

[4] Saver JL Goyal M Van der Lugt A Menon BK Majoie CB Dippel DW Time to treatment with endovascular thrombectomy and outcomes from ischemic stroke: a meta-analysis. JAMA (2016) 316:1279–88. 10.1001/jama.2016.13647 27673305

[5] Grotta JC Jacobs TP Koroshetz WJ Moskowitz MA. Stroke program review group an interim report. Stroke (2008) 39:1364–70. 10.1161/STROKEAHA.107.510776 18309142

[6] Zhang ZG Chopp M. Neurorestorative therapies for stroke: underlying mechanisms and translation to the clinic. Lancet Neurol (2009) 8:491–500. 10.1016/S1474-4422(09)70061-4 19375666 PMC2727708

[7] Bliss T Guzman R Daadi M Steinberg GK Cell transplantation therapy for stroke. Stroke (2007) 38:817–26. 10.1161/01.STR.0000247888.25985.62 17261746

[8] Hao L Zou Z Tian H Zhang Y Zhou H Liu L. Stem cell-based therapies for ischemic stroke. Biomed Res Int (2014) 2014:468748. 10.1155/2014/468748 24719869 PMC3955655

[9] Zakhem M Yawno T Jenkin G Miller S. Stem cell therapy to protect and repair the developing brain: a review of mechanisms of action of cord blood and amnion epithelial derived cells. Front Neurosci (2013) 7:194. 10.3389/fnins.2013.00194 24167471 PMC3807037

[10] Zents K Copray S. The therapeutic potential of induced pluripotent stem cells after stroke: evidence from rodent models. Curr Stem Cell Res Ther (2016) 11:166–74. 10.2174/1574888X10666150728121324 26216130

[11] Thomson JA Itskovitz-Eldor J Shapiro SS Waknitz MA Swiergiel JJ Marshall VS Embryonic stem cell lines derived from human blastocysts. Science (1998) 282:1145–7. 10.1126/science.282.5391.1145 9804556

[12] Wichterle H Lieberam I Porter JA Jessell TM. Directed differentiation of embryonic stem cells into motor neurons. Cell (2002) 110:385–97. 10.1016/S0092-8674(02)00835-8 12176325

[13] Nagai N Kawao N Okada K Okumoto K Teramura T Ueshima S Systemic transplantation of embryonic stem cells accelerates brain lesion decrease and angiogenesis. Neuroreport (2010) 21:575–9. 10.1097/WNR.0b013e32833a7d2c 20431496

[14] Tae-Hoon L Yoon-Seok L. Transplantation of mouse embryonic stem cell after middle cerebral artery occlusion. Acta Cirúrgica Brasileira (2012) 27:333–9. 10.1590/S0102-86502012000400009 22534809

[15] Toda H Takahashi J Iwakami N Kimura T Hoki S Mozumi-Kitamura K Grafting neural stem cells improved the impaired spatial recognition in ischemic rats. Neurosci Lett (2001) 316:9–12. 10.1016/S0304-3940(01)02331-X 11720766

[16] Ishibashi S Sakaguchi M Kuroiwa T Yamasaki M Kanemura Y Shizuko I Human neural stem/progenitor cells, expanded in long-term neurosphere culture, promote functional recovery after focal ischemia in Mongolian gerbils. J Neurosci Res (2004) 78:215–23. 10.1002/jnr.20246 15378509

[17] Polezhaev L Alexandrova M Transplantation of embryonic brain tissue into the brain of adult rats after hypoxic hypoxia. J Hirnforsch (1983) 25:99–106. 6725943

[18] Polezhaev L Alexandrova M Vitvitsky V Girman S Golovina I Morphological, biochemical and physiological changes in brain nervous tissue of adult intact and hypoxia-subjected rats after transplantation of embryonic nervous tissue. J Hirnforsch (1984) 26:281–9. 4031487

[19] Darsalia V Kallur T Kokaia Z. Survival, migration and neuronal differentiation of human fetal striatal and cortical neural stem cells grafted in stroke-damaged rat striatum. Eur J Neurosci (2007) 26:605–14. 10.1111/j.1460-9568.2007.05702.x 17686040

[20] Takahashi K Yasuhara T Shingo T Muraoka K Kameda M Takeuchi A Embryonic neural stem cells transplanted in middle cerebral artery occlusion model of rats demonstrated potent therapeutic effects, compared to adult neural stem cells. Brain Res (2008) 1234:172–82. 10.1016/j.brainres.2008.07.086 18703033

[21] Guzman R Bliss T De Los Angeles A Moseley M Palmer T Steinberg G. Neural progenitor cells transplanted into the uninjured brain undergo targeted migration after stroke onset. J Neurosci Res (2008) 86:873–82. 10.1002/jnr.21542 17975825

[22] Mine Y Tatarishvili J Oki K Monni E Kokaia Z Lindvall O. Grafted human neural stem cells enhance several steps of endogenous neurogenesis and improve behavioral recovery after middle cerebral artery occlusion in rats. Neurobiol Dis (2013) 52:191–203. 10.1016/j.nbd.2012.12.006 23276704

[23] Roitberg BZ Mangubat E Chen E-Y Sugaya K Thulborn KR Kordower JH Survival and early differentiation of human neural stem cells transplanted in a nonhuman primate model of stroke. J Neurosurg (2006) 105:96–102. 10.3171/jns.2006.105.1.96 16871883

[24] Honmou O Onodera R Sasaki M Waxman SG Kocsis JD. Mesenchymal stem cells: therapeutic outlook for stroke. Trends Mol Med (2012) 18:292–7. 10.1016/j.molmed.2012.02.003 22459358

[25] Sanberg PR Eve DJ Metcalf C Borlongan CV Advantages and challenges of alternative sources of adult-derived stem cells for brain repair in stroke. Prog Brain Res (2011) 201:99–117. 10.1016/B978-0-444-59544-7.00006-8 23186712

[26] Huang W Mo X Qin C Zheng J Liang Z Zhang C. Transplantation of differentiated bone marrow stromal cells promotes motor functional recovery in rats with stroke. Neurol Res (2013) 35:320–8. 10.1179/1743132812Y.0000000151 23485057

[27] Tohill M Mantovani C Wiberg M Terenghi G. Rat bone marrow mesenchymal stem cells express glial markers and stimulate nerve regeneration. Neurosci Lett (2004) 362:200–3. 10.1016/j.neulet.2004.03.077 15158014

[28] Liu Z Li Y Zhang L Xin H Cui Y Hanson LR Subacute intranasal administration of tissue plasminogen activator increases functional recovery and axonal remodeling after stroke in rats. Neurobiol Dis (2012) 45:804–9. 10.1016/j.nbd.2011.11.004 22115941 PMC3259280

[29] Yoo KH Jang IK Lee MW Kim HE Yang MS Eom Y Comparison of immunomodulatory properties of mesenchymal stem cells derived from adult human tissues. Cell Immunol (2009) 259:150–6. 10.1016/j.cellimm.2009.06.010 19608159

[30] Bang OY Lee JS Lee PH Lee G. Autologous mesenchymal stem cell transplantation in stroke patients. Ann Neurol (2005) 57:874–82. 10.1002/ana.20501 15929052

[31] Lee JS Hong JM Moon GJ Lee PH Ahn YH Bang OY. A long-term follow-up study of intravenous autologous mesenchymal stem cell transplantation in patients with ischemic stroke. Stem Cells (2010) 28:1099–106. 10.1002/stem.430 20506226

[32] Suárez-Monteagudo C Hernández-Ramírez P Álvarez-González L García-Maeso I de la Cuétara-Bernal K Castillo-Díaz L Autologous bone marrow stem cell neurotransplantation in stroke patients. An open study. Restor Neurol Neurosci (2009) 27:151–61. 10.3233/RNN-2009-0483 19531871

[33] Takahashi K Tanabe K Ohnuki M Narita M Ichisaka T Tomoda K Induction of pluripotent stem cells from adult human fibroblasts by defined factors. Cell (2007) 131:861–72. 10.1016/j.cell.2007.11.019 18035408

[34] Takahashi K Yamanaka S. Induction of pluripotent stem cells from mouse embryonic and adult fibroblast cultures by defined factors. Cell (2006) 126:663–76. 10.1016/j.cell.2006.07.024 16904174

[35] Ito D Okano H Suzuki N. Accelerating progress in induced pluripotent stem cell research for neurological diseases. Ann Neurol (2012) 72:167–74. 10.1002/ana.23596 22926850

[36] Abe K Yamashita T Takizawa S Kuroda S Kinouchi H Kawahara N. Stem cell therapy for cerebral ischemia: from basic science to clinical applications. J Cereb Blood Flow Metab (2012) 32:1317–31. 10.1038/jcbfm.2011.187 22252239 PMC3390814

[37] Chen S-J Chang C-M Tsai S-K Chang Y-L Chou S-J Huang S-S Functional improvement of focal cerebral ischemia injury by subdural transplantation of induced pluripotent stem cells with fibrin glue. Stem Cells Dev (2010) 19:1757–67. 10.1089/scd.2009.0452 20192839

[38] Kawai H Yamashita T Ohta Y Deguchi K Nagotani S Zhang X Tridermal tumorigenesis of induced pluripotent stem cells transplanted in ischemic brain. J Cereb Blood Flow Metab (2010) 30:1487–93. 10.1038/jcbfm.2010.32 20216552 PMC2949240

[39] Miura K Okada Y Aoi T Okada A Takahashi K Okita K Variation in the safety of induced pluripotent stem cell lines. Nat Biotechnol (2009) 27:743–5. 10.1038/nbt.1554 19590502

[40] Yamashita T Kawai H Tian F Ohta Y Abe K. Tumorigenic development of induced pluripotent stem cells in ischemic mouse brain. Cell Transplant (2011) 20:883–91. 10.3727/096368910X539092 21054935

[41] Baker EW Platt SR Lau VW Grace HE Holmes SP Wang L Induced pluripotent stem cell-derived neural stem cell therapy enhances recovery in an ischemic stroke pig model. Sci Rep (2017) 7:10075. 10.1038/s41598-017-10406-x 28855627 PMC5577218

[42] Fong CY Gauthaman K Bongso A. Teratomas from pluripotent stem cells: a clinical hurdle. J Cell Biochem (2010) 111:769–81. 10.1002/jcb.22775 20665544

[43] Ghosh Z Huang M Hu S Wilson KD Dey D Wu JC. Dissecting the oncogenic and tumorigenic potential of differentiated human induced pluripotent stem cells and human embryonic stem cells. Cancer Res (2011) 71:5030–9. 10.1158/0008-5472.CAN-10-4402 21646469 PMC3138859

[44] Fu W Wang SJ Zhou GD Liu W Cao Y Zhang WJ Residual undifferentiated cells during differentiation of induced pluripotent stem cells in vitro and in vivo. Stem Cells Dev (2011) 21:521–9. 10.1089/scd.2011.0131 21631153

[45] Sun N Longaker MT Wu JC. Human iPS cell-based therapy: considerations before clinical applications. Cell Cycle (2010) 9:880–5. 10.4161/cc.9.5.10827 20160515 PMC3638036

[46] Yang S Lin G Tan YQ Zhou D Deng LY Cheng DH Tumor progression of culture-adapted human embryonic stem cells during long-term culture. Genes Chromosomes Cancer (2008) 47:665–79. 10.1002/gcc.20574 18470900

[47] Asadi MH Mowla SJ Fathi F Aleyasin A Asadzadeh J Atlasi Y. OCT4B1, a novel spliced variant of OCT4, is highly expressed in gastric cancer and acts as an antiapoptotic factor. Int J Cancer (2011) 128:2645–52. 10.1002/ijc.25643 20824712

[48] Sholl LM Barletta JA Yeap BY Chirieac LR Hornick JL. Sox2 protein expression is an independent poor prognostic indicator in stage I lung adenocarcinoma. Am J Surg Pathol (2010) 34:1193. 10.1097/PAS.0b013e3181e5e024 20631605 PMC2923819

[49] Tian Y Luo A Cai Y Su Q Ding F Chen H MicroRNA-10b promotes migration and invasion through KLF4 in human esophageal cancer cell lines. J Biol Chem (2010) 285:7986–94. 10.1074/jbc.M109.062877 20075075 PMC2832949

[50] Albihn A Johnsen JI Henriksson MA. MYC in oncogenesis and as a target for cancer therapies. Adv Cancer Res (2010) 107:163–224. 10.1016/S0065-230X(10)07006-5 20399964

[51] Ruggero D. The role of Myc-induced protein synthesis in cancer. Cancer Res (2009) 69:8839–43. 10.1158/0008-5472.CAN-09-1970 19934336 PMC2880919

[52] Schoenhals M Kassambara A De Vos J Hose D Moreaux J Klein B. Embryonic stem cell markers expression in cancers. Biochem Biophys Res Commun (2009) 383:157–62. 10.1016/j.bbrc.2009.02.156 19268426

[53] Marión RM Strati K Li H Murga M Blanco R Ortega S A p53-mediated DNA damage response limits reprogramming to ensure iPS cell genomic integrity. Nature (2009) 460:1149–53. 10.1038/nature08287 19668189 PMC3624089

[54] Fong CY Peh GS Gauthaman K Bongso A Separation of SSEA-4 and TRA-1–60 labelled undifferentiated human embryonic stem cells from a heterogeneous cell population using magnetic-activated cell sorting (MACS) and fluorescence-activated cell sorting (FACS). Stem Cell Rev Rep (2009) 5:72–80. 10.1007/s12015-009-9054-4 19184635

[55] Choo AB Tan HL Ang SN Fong WJ Chin A Lo J Selection against undifferentiated human embryonic stem cells by a cytotoxic antibody recognizing podocalyxin-like protein-1. Stem Cells (2008) 26:1454–63. 10.1634/stemcells.2007-0576 18356574

[56] Dean SK Yulyana Y Williams G Sidhu KS Tuch BE. Differentiation of encapsulated embryonic stem cells after transplantation. Transplantation (2006) 82:1175–84. 10.1097/01.tp.0000239518.23354.64 17102769

[57] Savitz SI Dinsmore J Wu J Henderson GV Stieg P Caplan LR. Neurotransplantation of fetal porcine cells in patients with basal ganglia infarcts: a preliminary safety and feasibility study. Cerebrovasc Dis (2005) 20:101–7. 10.1159/000086518 15976503

[58] Smith EJ Stroemer RP Gorenkova N Nakajima M Crum WR Tang E Implantation site and lesion topology determine efficacy of a human neural stem cell line in a rat model of chronic stroke. Stem Cells (2012) 30:785–96. 10.1002/stem.1024 22213183

[59] Shen L Li Y Chopp M. Astrocytic endogenous glial cell derived neurotrophic factor production is enhanced by bone marrow stromal cell transplantation in the ischemic boundary zone after stroke in adult rats. Glia (2010) 58:1074–81. 10.1002/glia.20988 20468049 PMC3096459

[60] Zacharek A Chen J Cui X Li A Li Y Roberts C Angiopoietin1/Tie2 and VEGF/Flk1 induced by MSC treatment amplifies angiogenesis and vascular stabilization after stroke. J Cereb Blood Flow Metab (2007) 27:1684–91. 10.1038/sj.jcbfm.9600475 17356562 PMC2796470

[61] Chen J Li Y Wang L Lu M Zhang X Chopp M. Therapeutic benefit of intracerebral transplantation of bone marrow stromal cells after cerebral ischemia in rats. J Neurol Sci (2001) 189:49–57. 10.1016/S0022-510X(01)00557-3 11535233

[62] Bühnemann C Scholz A Bernreuther C Malik CY Braun H Schachner M Neuronal differentiation of transplanted embryonic stem cell-derived precursors in stroke lesions of adult rats. Brain (2006) 129:3238–48. 10.1093/brain/awl261 17018551

[63] De Feo D Merlini A Laterza C Martino G. Neural stem cell transplantation in central nervous system disorders: from cell replacement to neuroprotection. Curr Opin Neurol (2012) 25:322–33. 10.1097/WCO.0b013e328352ec45 22547103

[64] Chen L Zhang G Khan AA Guo X Gu Y. Clinical efficacy and meta-analysis of stem cell therapies for patients with brain ischemia. Stem Cells Int (2016) 2016:6129579. 10.1155/2016/6129579 27656217 PMC5021879

[65] Detante O Moisan A Hommel M Jaillard A. Controlled clinical trials of cell therapy in stroke: meta-analysis at six months after treatment. Int J Stroke (2017) 12(7):748–51. 10.1177/1747493017696098 28884654

[66] Moniche F Rosado-de-Castro PH Escudero I Zapata E de la Torre Laviana FJ Mendez-Otero R Increasing dose of autologous bone marrow mononuclear cells transplantation is related to stroke outcome: results from a pooled analysis of two clinical trials. Stem Cells Int (2016) 2016:8 10.1155/2016/8657173 PMC4972913 27525011

[67] Kalladka D Sinden J Pollock K Haig C McLean J Smith W Human neural stem cells in patients with chronic ischaemic stroke (PISCES): a phase 1, first-in-man study. Lancet (2016) 388:787–96. 10.1016/S0140-6736(16)30513-X 27497862

[68] Honmou O Phase III clinical trial using autologous mesenchymal stem cells for stroke patients. Nihon rinsho (2016) 74(4):649–54. 27333754

[69] Steinberg GK Kondziolka D Wechsler LR Lunsford LD Coburn ML Billigen JB Clinical outcomes of transplanted modified bone marrow-derived mesenchymal stem cells in stroke: a phase 1/2a study. Stroke (2016) 47(7):1817–24. 10.1161/STROKEAHA.116.012995 27256670 PMC5828512

[70] Nagpal A Kremer KL Hamilton-Bruce MA Kaidonis X Milton AG Levi C TOOTH (the open study of dental pulp stem cell therapy in humans): study protocol for evaluating safety and feasibility of autologous human adult dental pulp stem cell therapy in patients with chronic disability after stroke. Int J Stroke (2016) 11:575–85. 10.1177/1747493016641111 27030504

[71] Battistella V de Freitas GR da Fonseca LMB Mercante D Gutfilen B Goldenberg RC Safety of autologous bone marrow mononuclear cell transplantation in patients with nonacute ischemic stroke. Regen Med (2011) 6:45–52. 10.2217/rme.10.97 21175286

[72] Qiao L-Y Huang F-J Zhao M Xie J-H Shi J Wang J A two-year follow-up study of cotransplantation with neural stem/progenitor cells and mesenchymal stromal cells in ischemic stroke patients. Cell Transplant (2014) 23:S65–72. 10.3727/096368914X684961 25333752

[73] Yang B Migliati E Parsha K Schaar K Xi X Aronowski J Intra-arterial delivery is not superior to intravenous delivery of autologous bone marrow mononuclear cells in acute ischemic stroke. Stroke (2013) 44:3463–72. 10.1161/STROKEAHA.111.000821 24114454 PMC4209211

[74] Bakondi B Shimada IS Perry A Munoz JR Ylostalo J Howard AB CD133 identifies a human bone marrow stem/progenitor cell sub-population with a repertoire of secreted factors that protect against stroke. Mol Ther (2009) 17:1938–47. 10.1038/mt.2009.185 19690521 PMC2835040

[75] Hess DC Wechsler LR Clark WM Savitz SI Ford GA Chiu D Safety and efficacy of multipotent adult progenitor cells in acute ischaemic stroke (MASTERS): a randomised, double-blind, placebo-controlled, phase 2 trial. Lancet Neurol (2017) 16:360–8. 10.1016/S1474-4422(17)30046-7 28320635

[76] Muir KW. Clinical trial design for stem cell therapies in stroke: what have we learned? Neurochem Int (2017) 106:108–13. 10.1016/j.neuint.2016.09.011 27623094

